# The Common Task

**Published:** 1907-08-24

**Authors:** 


### THE COMMON TASK.

**Correspondence and Queries** for this section should be sent to the Editor of The Hospital, 28 Southampton Street, Strand, London, and marked "Nursing Administration."

### The Shortage of Probationers in America.

There seems some reason to fear that women are being deterred from nursing in those States where Registration has been adopted, by the preliminary educational requirements, and by the examinations imposed before the certificates of registration are issued. Many women who can make a very good appearance when examined in their own school under familiar conditions flinch and fail when subjected to the ordeal of a public and formal examination whether written or *viva voce*, and some recoil from it altogether. It is certain that registration is not performing all the wonders it was warranted to achieve, and much may be learned by attentively watching the impediments to its progress which are being faced with undaunted courage by its advocates on the other side of the Atlantic. It seems as if the American nurse was becoming restive at the three years' course, and we are glad that this difficulty has at any rate so far not made itself felt in this country.

### Petty Cash.

The command of a little ready money often facilitates the housekeeper's economies to an extent "red tape" declines to recognise. For this reason the matron whose duties include the catering and the purchase of household necessaries ought to be allowed the use of petty cash with which to seize opportunities. "Can nothing ever be bought cheap for a workhouse?" a matron was heard lately to complain. She needed some trifle, which she could have procured at small cost, but was compelled to apply for it under the regulated conditions, and when after long delay it had passed through the various stages which authorised its purchase the result was the most costly article of its kind which it was possible to procure. During the present glut of fruit in London the matron with the necessary means could make enough jam for the whole year at half the cost of the ordinary adulterated shop article, but in most institutions it would be unheard of for any deviation to take place from the usual course of buying from one regular dealer, at "institution prices."

### The Ethics of Matronship.

A useful little book on "Open Doors for Irishwomen" has been issued by the Irish Bureau for the Employment of Women, and it is satisfactory to find that elaborate needlework no longer stands out as the principal if not the only source of income for the Irish lady debarred from living on her "rentes." Nursing plays a conspicuous part among the occupations recommended, and among the various branches of work we note, too, "Matronships of Institutions." Under this heading a few maxims are given, from which we quote the following:—"Never seem in a hurry, never decide hurriedly, and once decided never change." Now the very essence of superintendence is that the head is called on all day long to decide "in a hurry," generally, it is true, about trifles, but often about more important things. She is compelled to train herself to be prompt, and however seemly it may be that she should "never seem in a hurry," she can no more help often having to work at top speed than she can insist upon being allowed to deliberate when some frequent emergency arises. Moreover, "once dedecided, never change," is at the root of that dense officialism from which in England we are more free than in the bureaucratic countries of Europe. Why, new facts may transpire, conditions swiftly be reversed, judgment, however deliberate, be at fault. There is no weakness so deadly as the weak obstinacy of the woman who is afraid of being thought weak.

### Colonial Nursing Association.

In no direction has central organisation proved more beneficial than in the spread of trained nursing along the outer borders of the Empire. Fifteen, or even ten, years ago the woman who ventured to take up work in Africa or in the East was adventurous or self-sacrificing in the extreme. Her fitness for the work was conjectural, her prospects undefined, her position on arrival ambiguous. Splendid pioneer work was done under conditions of the most adverse description, but the wastage of health and energy was discouraging even to those who saw most clearly the need for the trained nurse in districts where white men were in a small minority, and a prey to every kind of disease. All this has been altered in the course of a few years only by the Colonial Nursing Association. During the ten years of its existence the Association has sent out no fewer than 344 nurses for Government service and private work, and in each case the greatest care has been observed in selecting women of the right type, well trained, physically fit, and suited by temperament for the exceptional difficulties of the posts they have been called on to fill. Instead of going to unknown dangers the nurses of the Association are safeguarded right along the line. Favourable conditions as regards salary, leave, and passage money are secured to them by the action of the Association, proper quarters are engaged for them, and by working whenever possible through the medium of the Colonial Governments, their position is assured from the moment they set foot in their new sphere of work. An immense field of occupation has thus been thrown open, a field as yet but partially tilled, and capable of almost boundless development. The wisdom which has guided the counsels of the Association in steering a straight course through manifold perplexities of administration can be best appreciated by those acquainted with the regions for whom the nurses are required. It is gratifying to know that the matrons and nurses sent out under this admirable system are contributing in no small degree to the improvements now taking place in the hospitals throughout the Crown Colonies and Protectorates.

